# A 6 years trends in hospitalization: retrospective study of Traumatic Brain Injury

**DOI:** 10.1093/eurpub/ckac131.418

**Published:** 2022-10-25

**Authors:** N Chikhladze, E Burkadze, N Pitskhelauri, A Tsiskaridze, C Peek-Asa, M Coman

**Affiliations:** 1 Faculty of Medicine, Ivane Javakhishvili Tbilisi State University, Tbilisi, Georgia; 2 University of California, San Diego, USA; 3 Department of Public Health, Babes-Bolyai University, Cluj-Napoca, Romania

## Abstract

**Introduction:**

Traumatic Brain Injury (TBI) is a significant public health issue globally, and the mortality and morbidity burden is particularly high in low- and middle-income counties (LMICs). The WHO predicts a disproportionately large increase of TBI burden in LMICs. The aim of this study was to identify trends in hospitalization associated with TBI in Georgia from 2015-2020.

**Methods:**

The study was designed in the framework of the project INITIatE: International Collaboration to Increase TBI Surveillance in Europe, funded by the US National Institutes of Health (NIH/NINDS R21NS098850). The surveillance database of National Center for Disease Control and Public Health of Georgia was used for the study.

**Results:**

During the study period a total 51 147 patients were admitted in hospitals throughout the country. In 2015-2019 the hospitalization increased and highest number of cases was in 2019 (n = 11779; 23,0%), in 2020 hospitalization decreased in comparison with the previous year (n = 9228; 18,0%). The highest number of burn injuries (n = 22963; 45,0%) occurred in the capital of Georgia (Tbilisi). Among hospitalized patients about 61,0% were males (n = 31162) and 39,0% females (n = 19985), retrospectively with ratio 1,6:1. The modal age of hospitalized patients was 25-44 and the highest hospitalization was in the age group of 15-24. 92,5% of cases were unintentional. The leading cause of Traumatic Brain Injury in all years were falls with some variations (57%-71%), followed by road traffic injuries (12%-25%). The average of LOS was 3 days, the highest LOS was 702 days. 1,6% (n = 805) of patients died. The most common mechanism of fatal injuries were falls.

**Conclusions:**

The study provide important information about trends in hospitalization, size of the TBI problem, which is crucial for elaborating relevant policy and establishing priorities in order to reduce the burden of Traumatic Brain Injury in Georgia, as well to identify directions for further TBI related research.

**Key messages:**

• Epidemiological data are essential for designing relevant preventive programs.

• Prevention is a key component of public health efforts to reduce TBI burden.

